# Karyotypes of three species of *Hyperophora* Brunner von Wattenwyl, 1878 (Tettigoniidae, Phaneropterinae) enable morphologically similar species to be distinguished

**DOI:** 10.3897/CompCytogen.v13i1.31803

**Published:** 2019-04-02

**Authors:** Bruno Cansanção Silva, Lucas Henrique Bonfim Souza, Juliana Chamorro-Rengifo, Douglas Araujo

**Affiliations:** 1 Programa de Pós-Graduação em Biologia Animal, Instituto de Biociências, Universidade Federal de Mato Grosso do Sul, UFMS, Cidade Universitária, 79070-900, Campo Grande, Mato Grosso do Sul, Brazil Universidade Federal de Mato Grosso do Sul Campo Grande Brazil; 2 Setor de Biologia Geral, Instituto de Biociências, Universidade Federal de Mato Grosso do Sul, UFMS, Cidade Universitária, 79070-900, Campo Grande, Mato Grosso do Sul, Brazil Universidade Federal de Mato Grosso do Sul Campo Grande Brazil

**Keywords:** Aniarae, fluorescent *in situ* hybridization, meiosis, Pantanal

## Abstract

Phaneropterinae is the largest subfamily of Tettigoniidae, distributed across the globe. There are few cytogenetic studies regarding this group, as in the case of the genus group Aniarae, which represents only two karyotyped species. The current study aims to analyze cytogenetically three species of *Hyperophora* Brunner von Wattenwyl, 1878 from Brazil. The male diploid number of *Hyperophoraminor* Brunner von Wattenwyl, 1891 and *Hyperophoramajor* Brunner von Wattenwyl, 1878 is 2n♂= 31, whereas *Hyperophorabrasiliensis* Brunner von Wattenwyl, 1878 has shown 2n♂= 29. These three species possess an X0 sex chromosome system and telo/acrocentric chromosome morphology. The only species found in the Pantanal biome, *H.brasiliensis*, can be chromosomally distinguished from the Cerrado biome species *H.major* and *H.minor*, due to the difference in chromosome number (2n♂= 29 and 2n♂= 31, respectively).

## Introduction

Tettigoniidae comprise 7598 species distributed worldwide, 2634 of them belonging to Phaneropterinae, the largest subfamily of the group. The genus *Hyperophora* Brunner von Wattenwyl, 1878 includes 16 South American species and belongs to Aniarae, along with other six genera (*Aniarella* Bolívar, 1906, *Burgilis* Stål, *Corymeta* Brunner von Wattenwyl, 1878, *Coryphoda* Brunner von Wattenwyl, 1878, *Pseudoburgilis* Brunner von Wattenwyl, 1878 and *Tetana* Brunner von Wattenwyl, 1878) (Eades et al. 2018).

For some *Hyperophora* species there is a paucity of descriptive information regarding the intraspecific morphological variations. Rehn (1907) described a male of the species *Hyperophoramajor* Brunner von Wattenwyl, 1878, commenting that the individual is smaller than those described by Brunner von Wattenwyl (1878) and published a schematic of the male cercus along with a sketch of *Hyperophorabrasiliensis* Brunner von Wattenwyl, 1878 cerci apparently based on individuals different from the type material. The drawings of the cerci of *H.brasiliensis* and *H.major* are slightly similar, raising doubts as to whether they are simply morphological variations, since as previously reported there is no complete description that presents other robust characteristics that allow an accurate identification.

Cytogenetic data regarding Tettigoniidae are scarce (Warchałowska-Śliwa 1998). Within Phaneropterinae, at least 160 species were karyotyped (Warchałowska-Śliwa et al. 2011) and the most studied taxa belong to the tribe Barbistini, with more than 50 analyzed species (Warchałowska-Śliwa et al. 2013). Karyological studies in Phaneropterinae showed that the diploid number ranged from 16 to 33 in males, predominantly with the ♂X0/♀XX Sex Chromosome System (SCS) and telo/acrocentric chromosomes. Despite this variation, the most common diploid number for the subfamily is 2n♂= 31 and therefore it is likely to be the Phaneropterinae ancestor karyotype (White 1973, Warchałowska-Śliwa 1998, Hemp et al. 2014).

*Aniarellaferraciui* Piza, 1977 and *Hyperophoraangustipennis* Brunner von Wattenwyl, 1891 are the only species of the whole Aniarae group that were chromosomally analyzed, presenting 2n♂= 21, X0 and 2n♂= 31, X0, respectively (Ferreira 1976, Ferreira and Mesa 2007).

In this work, we describe the karyotype of *Hyperophorabrasiliensis* Brunner von Wattenwyl, 1878, *Hyperophoramajor* and *Hyperophoraminor* Brunner von Wattenwyl, 1891, to discuss the chromosomal evolution and the cytotaxonomy of the group.

## Material and methods

The specimens were collected at two localities in the state of Mato Grosso do Sul (MS), Brazil, from November 2015 to February 2017 and were deposited in the Coleção Zoológica de Referência da UFMS (ZUFMS) with the exception of one male specimen of *H.major*, which was used in the work of Serrão et al. (2018) (Table 1).

**Table 1.** Collection data. Site, number and sex of specimens, voucher numbers, and number of analyzed cells of the *Hyperophora* species cytogenetically examined in this study.

Species	Collection site	Specimens	Voucher (ZUFMS)	Number of cells
*Hyperophorabrasiliensis* Brunner von Wattenwyl, 1878	Base de Estudos do Pantanal (BEP), municipality of Corumbá [19°34'37"S, 57°00'42"W]	2♂/1♀	ZUFMS-ORTO710; ZUFMS-ORTO711; ZUFMS-ORTO712	67
*Hyperophoramajor* Brunner von Wattenwyl, 1878	Estância Sossego, municipality of Campo Grande [20°29'19.09"S, 54°39'39.06"W]	3♂/1♀	ZUFMS-ORT00713; ZUFMS-ORT00715; ZUFMS-ORT00716	61
*Hyperophoraminor* Brunner von Wattenwyl, 1891	Estância Sossego, municipality of Campo Grande [20°29'19.09"S, 54°39'39.06"W]	1♂/2♀	ZUFMS-ORT00714; ZUFMS-ORT00717; ZUFMS-ORT00718	100

The individuals were anesthetized in ether, dissected and fixed in 70% ethanol, with the exception of the gonads, which were used for chromosomal preparations and Giemsa staining following the procedures of Araujo et al. (2008). Slides from all three species were submitted to Fluorescence *in situ* hybridization (FISH) using the telomeric probe. This process employs a peptidic nucleic acid (PNA) (AATCC)_3_ probe (PNA Bio, Inc) that is complementary to the typical (TTAGG)_n_ telomeric repeats of Orthoptera, labeled with Alexa fluor 488 (ThermoFisher Scientific). It was followed the method of Genet et al. (2013), with a hybridization time of four hours at 37 °C, without heat denaturing, and mounted with ProLong Diamond antifade containing DAPI (ThermoFisher Scientific).

All cells were photographed using a Zeiss Axioimager D2 microscope with a monochromatic AxioCam 503 camera, employing the ZEN Pro software. Chromosome morphology was determined using the free software IMAGEJ (Rasband 1997–2018) and the LEVAN plugin (Sakamoto and Zacaro 2009), according to Levan et al. (1964) and Green and Sessions (1991), using respectively ten, nine and 18 mitotic metaphases of *H.brasiliensis*, *H.major* and *H.minor*.

## Results

**Figure 1.**
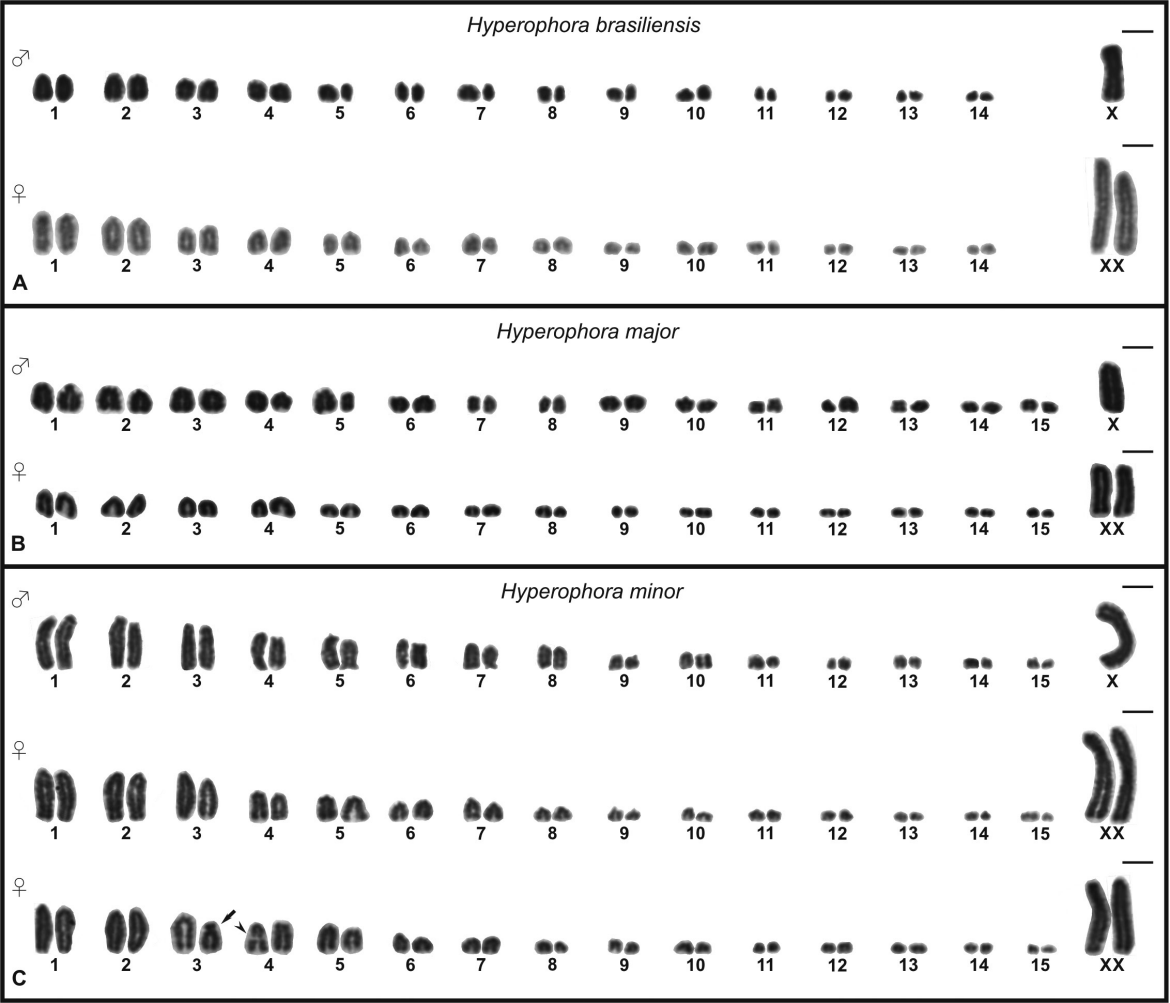
Karyotype of three *Hyperophora* species. **A***Hyperophorabrasiliensis* showing 2n♂=29 and 2n♀=30 **B***Hyperophoramajor* with 2n♂=31 and 2n♀=32 **C***Hyperophoraminor* exhibit 2n♂=31 and 2n♀=32. Arrow = heteromorphic chromosome. Arrowhead = heteropycnotic negative region. Scale bars: 5 μm.

**Figure 2.**
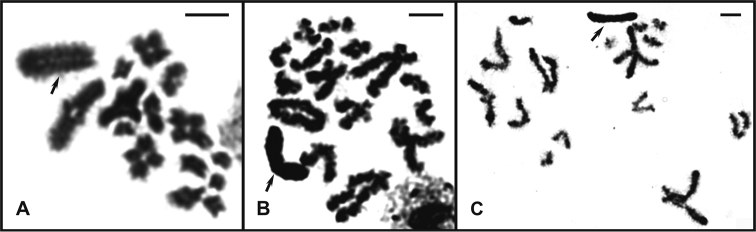
Male diplotenes of three *Hyperophora* species. **A***Hyperophorabrasiliensis* with 14II+X **B***Hyperophoramajor* showing 15II+X **C***Hyperophoraminor* exhibit 15II+X. Arrows = X chromosome. Scale bars: 5 μm.

*Hyperophorabrasiliensis* showed 2n♂= 29 and 2n♀= 30 (Fig. 1a). Spermatocytes I in diplotene exhibit 14 autosomal bivalents and one positively heteropycnotic sex univalent (Fig. 2a). Both *H.major* and *H.minor* presented 2n♂= 31 and 2n♀= 32 (Fig. 1b and c), however, only *H.minor* possess an interstitial heteropycnotic negative region in one telo/acrocentric chromosome of medium size (not visible in all cells due to chromosome condensation degree) (Fig. 1c). Male diplotene cells of these species exhibit 15 autosomal bivalents and one positively heteropycnotic sex univalent (Fig. 2b, c).

All three species possess the SCS of the type ♂X0/♀XX and showed exclusively telo/acrocentric chromosomes (Fig. 1), with the exception of one specimen of *H.minor*, which exhibited one submetacentric chromosome in all of the nine analyzed cells. (Fig. 1c).

**Figure 3.**
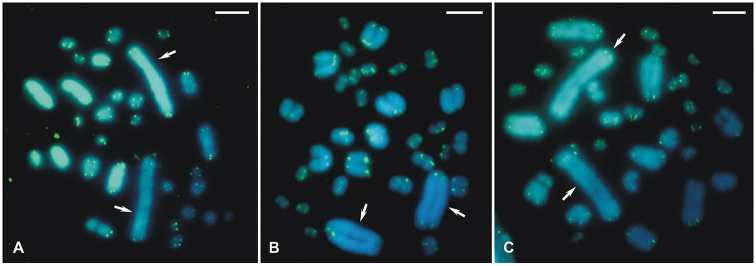
Female metaphases of three *Hyperophora* species with telomeric fluorescent *in situ* hybridization. **A***Hyperophorabrasiliensis* exhibit 2n♀=30 **B***Hyperophoramajor* with 2n♀=32 **C***Hyperophoraminor* showed 2n♀=32. Arrows = X chromosome. Scale bars: 5 μm.

Only the telomeric regions of all chromosomes were hybridized in the three species analyzed (Fig. 3). No interstitial telomeric sites (ITS) were observed in any of the cells submitted to telomeric FISH.

## Discussion

The karyotype composed of 2n♂= 31 and ♂X0/♀XX SCS, presenting only telo/acrocentric chromosomes found in *Hyperophoramajor* and *Hyperophoraminor* is the most common for katydids (Ferreira 1976, Warchałowska-Śliwa et al. 2011) and it was the same karyotype configuration presented in a congeneric species, *H.angustipennis*, the only *Hyperophora* species citogenetically analyzed up to now (Ferreira 1976). The heteromorphic pair found in one female of *H.minor* is the first recorded in Phaneropterinae, likely a result of one pericentric inversion.

*Hyperophorabrasiliensis* showed 2n♂= 29, that is, one autosomal pair less when compared to the other *Hyperophora* species and the most common pattern in Tettigoniidae (2n♂= 31). In Phaneropterinae, karyotypes with 2n♂= 29 and 2n♂= 31 within the same genus were found in *Holochlora* Stål, 1873, *Phaneroptera* Serville, 1831, and *Scuderia* Stål, 1873 (Warchałowska-Śliwa 1998). Thus, a reduction of one chromosomal pair, probably due to *in tandem* fusion, appears to occur independently several times within Phaneropterinae (Warchałowska-Śliwa 1998, Hemp et al. 2010). Despite the suggested chromosome fusion, no interstitial telomeric sites (ITS) were detected, which can reflect an ancient fusion event, that the telomeric region of the fused element was lost during the rearrangement, or that it is below the limit of FISH technique.

Interestingly, both species which showed 2n♂= 31 are sympatric in the Cerrado of Campo Grande, while *H.brasiliensis* (2n♂= 29), which was not registered in Campo Grande, was collected in the Pantanal of Corumbá (~ distance 270 Km). The cerci of *H.minor* differ enormously from the cerci of *H.brasiliensis* and *H.major*, thus permitting a rapid and accurate morphological identification of *H.minor*. In this study, it was determined that despite the morphological similarity of the cerci of *H.brasiliensis* and *H.major* (Brunner von Wattenwyl 1878), the karyotypes of *H.minor* and *H.major* are more similar to each other than those of *H.brasiliensis*, helping to distinguish these species.

Regarding the Aniarae group, all four *Hyperophora* species karyotyped (Ferreira 1976, present study) exhibited higher diploid numbers (2n♂= 31 or 2n♂= 29) than the only *Aniarella* species karyotyped up to now (2n♂= 21) (Ferreira and Mesa 2007). Differences of 10 or more chromosomes within karyotypes of closely related genera are uncommon among Phaneropterinae groups. However, the genus group Phyllopterae, *Itarissa* sp. presented 2n♂= 17, while *Phyllopterafosteri* Caudell, 1906 (cited as *Phyllopteramodesta* Piza, 1961) and *Phylloptera* sp. evidenced 2n♂= 31 (Ferreira 1977, Ferreira and Mesa 2007). In both cases, the species belong to a “genus group”, not a tribe. Genus groups are unreliable since there are not strict systematic studies supporting them. The clusters are allocated due to morphological similarities that could indicate the lack of a close kinship between the genera.

## Conclusion

The diploid number was useful in order to distinguish on chromosome level the species of *Hyperophora* from the Pantanal of those from other localities. The external morphological appearance is not directly related to similarity in the chromosome number for *Hyperophora*. Further research of other species of the Aniarae group is fundamental for assessing karyotype patterns within the clade, however, it is possible to affirm that the reduction from 2n♂=31 to 2n♂=29 is a recurrent event in Phaneropterinae.
